# Supplementary material for: Collateral sensitivity constrains resistance evolution of the CTX-M-15 β-lactamase
Source: Nat Commun. 2019 Feb 6;10:618. doi: 10.1038/s41467-019-08529-y (PMC6365502; doi:10.1038/s41467-019-08529-y)
Supplement: Supplementary file 1 — Supplementary Information [file 41467_2019_8529_MOESM1_ESM.pdf]

Collateral sensitivity constrains the resistance evolution of the CTX-M-15 β-lactamase

Rosenkilde and Munck et al.

Supplementary information

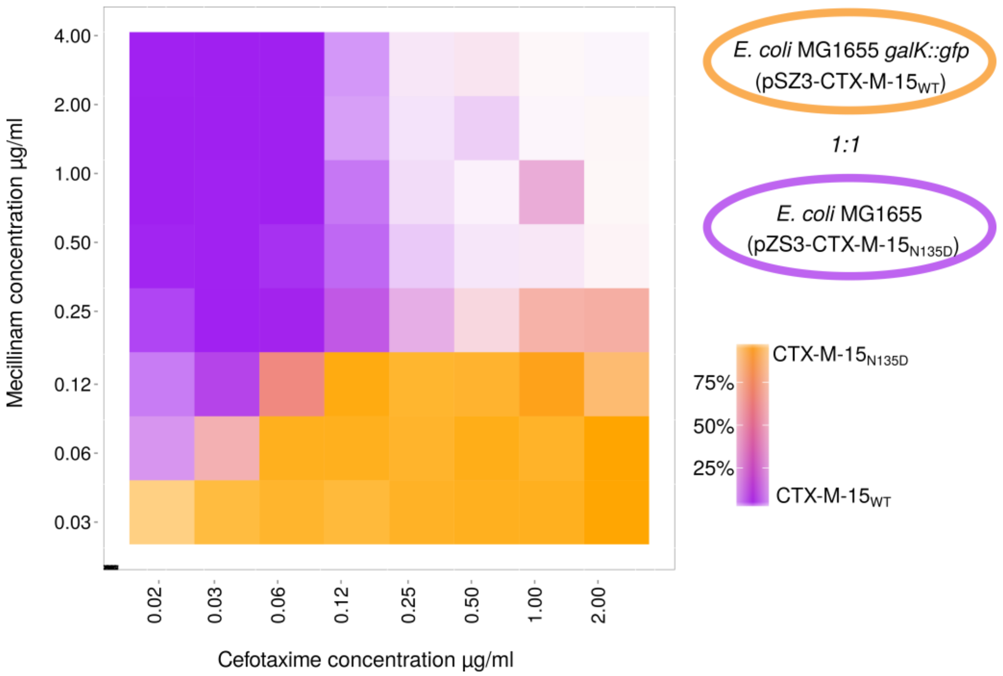

Supplementary Figure 1. 2D growth of CTX-M-15$_{N135D}$ and CTX-M-15$_{WT}$. A GFP tag was placed on *E. coli* MG1655 expressing either CTX-M-15$_{WT}$ or CTX-M-15$_{N135D}$ to verify that the GFP tag did not influence the growth results. *E. coli* MG1655 *galK*::*gfp* (pZS3-CTX-M-15$_{WT}$) was mixed with *E. coli* MG1655 (pZS3-CTX-M-15$_{N135D}$) in a 1:1 ratio and grown in a 2D gradient of mecillinam versus cefotaxime. Each square represents a well, and the colour represents the amount of either labelled CTX-M-15$_{N135D}$ (orange) or non-labelled CTX-M-15$_{WT}$ (purple). Darker colour indicates higher total cell count and lighter colour indicate lower cell count. The highest concentrations corresponded to mecillinam 4x MIC for CTX-M-15$_{WT}$ and cefotaxime 2x MIC for CTX-M-15$_{N135D}$.

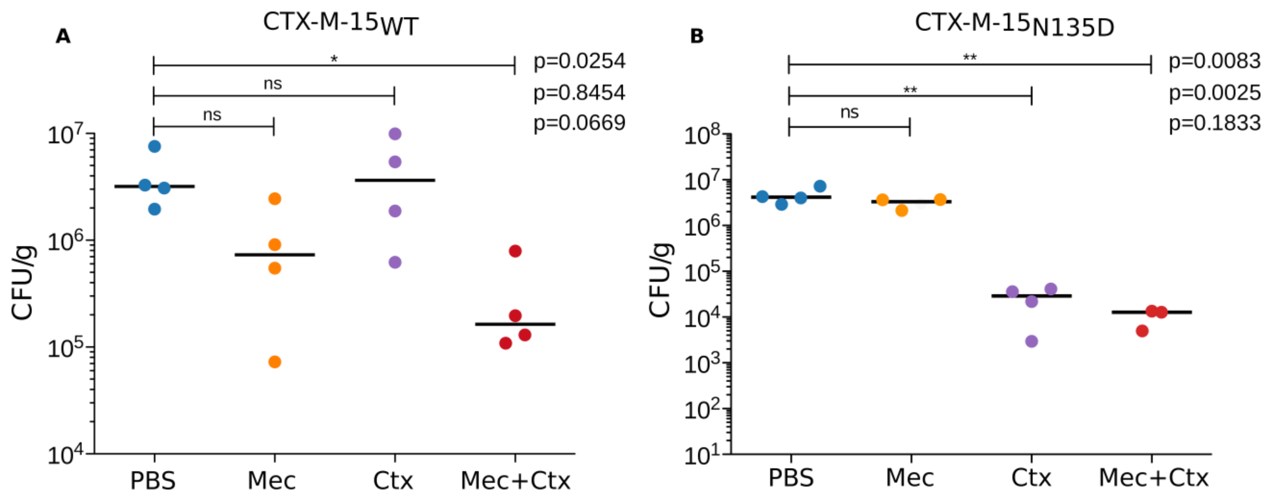

Supplementary Figure 2. Bacterial counts from mice infected with CTX-M-15$_{WT}$ or CTX-M-15$_{N135D}$. Mice infected via intraperitoneal injection with *S.* Typhimurium expressing either CTX-M-15$_{WT}$ strain (A) or the mutant strain carrying CTX-M-15$_{N135D}$ (B) were treated with mecillinam (Mec), cefotaxime (Ctx), or a combination of the two. PBS was used as a negative control. Bacterial counts from spleen (colony-forming units (CFU)/g) are shown (4 mice per group). Inoculum size was 1.5×10^5 CFU/mouse. ns: not significant; *$p < 0.05$; **$p < 0.01$. Horizontal bold line denotes the median. Statistical analysis was performed using unpaired Student's *t* test. One mouse infected with the mutant CTX-M-15$_{N135D}$ and treated with mecillinam in combination with cefotaxime cleared the infection. Data from this mouse are not plotted.

Strain	pipTZB	Mecillinam	mecTZB	mecCLA	amoCLA	Cefotaxime	Meropenem	Piperacillin
pZS3 empty vector	0.6	0.19	0.88	1.4	6.8	<2	0.07	22
CTX-M-15 WT	1.6	0.3	0.9	1.4	14	23	0.07	879
S133G	13	0.2	0.5	1.4	104	<2	0.07	472
N135D	1.3	15	2.9	5.5	7	<2	0.08	156
G239S	7.3	0.4	0.4	1.4	19	<2	0.08	208
S133G+N135D	12	0.3	1	1.8	18	<2	0.07	482
S133G+G239S	0.7	0.5	1.4	2.7	7.7	<2	0.07	31
N135D+G239S	0.7	0.19	1.3	1.3	7	<2	0.07	23.5
Clinical breakpoint	8	8	ND	ND	8	2	8	16

Supplementary Table 1. MIC values (µg/ml) for the CTX-M-15$_{WT}$ and CTX-M-15 mutants. All MIC values are based on expression in *E. coli* TOP10 and given in µg/ml. Ratios of inhibitor to antibiotic were as follows (concentrations in parenthesis is the concentration at 1x MIC for CTX-M-15$_{WT}$ and concentrations of inhibitor is listed after): piperacillin-tazobactam (pipTZB), 8:1 (4 µg/ml); mecillinam-tazobactam (mecTZB), 1:2 (0.25/0.44 µg/ml) ; mecillinam-clavulanic acid (mecCLA), 1:6.4 (0.25/1.6 µg/ml); amoxicillin-clavulanic acid (amoCLA), 4:1 (8 µg/ml), concentrations for drugs without inhibitor (at 1x CTX-M-15$_{WT}$ MIC): mecillinam: 0.25 µg/ml, meropenem: 0.06 µg/ml, piperacillin (256 µg/ml): ND: no data on the clinical breakpoint for this antibiotic-inhibitor combination. Clinical breakpoint source: clincalc.com/eucast/

MIC	PIP-TZB	Mecillinam	Meropenem	Ceftazidime
0.5	Lawn	Lawn	>1000	-
0.75	Lawn	>1000	0	-
1	Lawn	92	0	-
2	>1000	0	0	>1000
4	5	0	0	No cells

Supplementary Table 2. CFU count of CTX-M-15$_{WT}$ screened on various antibiotics. Numbers correspond to CFU on a plate and is an average of 2 replicate plates. No cells, no cells grew on this plate -, not tested. Lawn represents a plate covered in indistinct colonies, ~, CFU was estimated. PIP-TZB: piperacillin-tazobactam. Ceftazidime: positive control

MIC	PIP-TZB	Mecillinam	Meropenem	Ceftazidime
0.5	Lawn	Lawn	>1000	-
0.75	Lawn	>1000	0	-
1	Lawn	>1000	0	-
2	>1000	~1000	0	>1000
4	~1000	~1000	0	~100

Supplementary Table 3. CFU count of CTX-M-15 mutant library screened on various antibiotics. Numbers correspond to CFU on a plate and is averaged over 4 replicate plates. Mutant library Mut2Vol2 was used. -, not tested. Lawn represents a plate covered in indistinct colonies, ~, CFU was estimated. PIP-TZB: piperacillin-tazobactam. Ceftazidime: positive control

Primer name	Sequenced	Purpose
CTX-M-15-F	AAAGTCGAC-ATGGTTAAAAAATCACTGCG	CTX_M-15 amplification
CTX-M-15-R	AAAAGCTT-TTACAAACCGTCGGTGACG	CTX_M-15 amplification
gblok-CTX-M-15-F	GAGGAGGTAAAAGAGGTCG	Error-prone PCR
gblok-CTX-M-15-R	GCAGGAATTCGATATCAAGC	Error-prone PCR
pZ-insert-F	GCGAAACGATCCTCATCC	Geneblock amplification
CM-insert-NotI-R	CGCCGCAGCCGAACG	Geneblock amplification
AscI-CTX-M-15-F	AAAGTTAAAC-GAGGAGGTAAAAGAGGTCG	Amplification of error-prone PCR
PmeI-CTX-M-15-R	AAAGGCGGCGCC-GCAGGAATTCGATATCAAGC	Amplification of error-prone PCR
Ancestral-pZS3-F	CATCCCCCCTAGCATAAC	Amplification of pZS3 bb
Ancestral-pZS3-R	ATATTATTGAAGCATTTATCAGG	Amplification of pZS3 bb
PmeI-Ancestral-pZS3-F	AAAGTTTAAAC-CATCCCCCCTAGCATAAC	Amplification of pZS3 bb
AscI-Ancestral-pZS3-R	AAAGGCGCGCC-ATATTATTGAAGCATTTATCAGG	Amplification of pZS3 bb
Mut397F	5’P CGCTACAGTACGGCGATAAC 3’	Creation of mutant
Mut397R	5’ CGGCCGCGCTAAGCT 3’	Creation of mutant
Mut403F	5’P GATGACGTGGCGATGA 3’	Creation of mutant
Mut403R	5’ GCTGTACTGTAGCGCG 3’	Creation of mutant
Mut715F	5’P GGGATAAAACCAGCAG 3’	Creation of mutant
Mut715R	5’ CCACAACCCAGGAAGC 3’	Creation of mutant
Chee-pZ-seq-F	CAAATATGTATCCGCTCATG	Illumina sequencing
Chee-pZ-seq-R	AAAAAACCCCTCAAGACCC	Illumina sequencing

Supplementary Table 4. Primers used in this study
