# Supplementary material for: Collateral sensitivity constrains resistance evolution of the CTX-M-15 β-lactamase
Source: Nat Commun. 2019 Feb 6;10:618. doi: 10.1038/s41467-019-08529-y (PMC6365502; doi:10.1038/s41467-019-08529-y)
Supplement: Supplementary file 5 — Description of Additional Supplementary Files [file 41467_2019_8529_MOESM5_ESM.pdf]

Description of Additional Supplementary Files

File Name: Supplementary Data 1
Description: The complete sequence of the *bla*$_{CTX\text{-}M\text{-}15}$ gene and the β-lactamase promoter.
